# Hypothesis: cell signalling influences age-related risk of colorectal cancer

**DOI:** 10.1111/jcmm.12366

**Published:** 2014-11-11

**Authors:** Michael Bordonaro, Darina L Lazarova

**Affiliations:** Department of Basic Sciences, The Commonwealth Medical CollegeScranton, PA, USA

**Keywords:** colorectal cancer, Wnt, progerin, klotho, mTOR, rapamycin, senescence, ageing

## Abstract

We propose that ageing is linked to colonic carcinogenesis through crosstalk between Wnt activity and signalling pathways related to ageing and senescence: progerin, klotho and mTOR. Mutations in the Wnt signalling pathway are responsible for the majority of colorectal cancers (CRCs); however, hyperactivation of Wnt signalling by butyrate, a breakdown product of dietary fibre, induces CRC cell apoptosis. This effect of butyrate may in part explain the protective action of fibre against CRC. Hutchinson–Gilford progeria syndrome is a premature ageing disorder caused by accumulation of the progerin protein; however, healthy individuals also produce progerin in the course of their normal ageing. Progerin activates expression of the Wnt inhibitors HES1 and TLE1. Thus, we hypothesize that with age, the increasing expression of progerin suppresses butyrate-mediated Wnt hyperactivation and apoptosis, leading to increased CRC risk. Wild-type klotho contributes to a significantly increased lifespan; however, *Klotho* gene variants differ significantly between newborns and elderly. Klotho inhibits basal Wnt signalling activity; thus, the protein may function as a tumour suppressor for CRC. However, similar to progerin, klotho variants associated with lifespan differences may repress butyrate-mediated Wnt hyperactivation, and thus increase the risk of CRC. Finally, mTOR signalling has also been linked to human ageing, and crosstalk between Wnt and mTOR signalling may influence colonic tumourigenesis. Understanding how progerin, klotho and mTOR link ageing with colonic neoplastic development may lead to novel preventive and therapeutic strategies against CRC associated with age.

## General hypothesis

The risk of sporadic colorectal cancer (CRC) increases with age, a pattern shared with other neoplasms. The association between ageing and cancer is in part explained by the accumulation of mutations over time. However, crosstalk between Wnt signalling and signalling pathways related to cellular senescence may also contribute to colonic tumourigenesis. We propose that human ageing is linked to colonic carcinogenesis through crosstalk between Wnt activity and senescence signalling pathways mediated by progerin, klotho and mTOR.

## Wnt signalling, Notch activity and CRC

Wnt signalling is induced by the binding of Wnt ligands to their cell surface receptors, resulting in accumulation of dephosphorylated beta-catenin, which interacts with Tcf/Lef DNA binding proteins [1]. Beta-catenin transcriptional complexes are detected by their ability to drive transcription from promoters with Tcf/Lef consensus sites, influencing the expression of Wnt target genes [2,3]. Wnt activity is deregulated in the majority of CRCs because of mutations in genes such as *adenomatous polyposis coli* (*APC)* and *beta-cat*enin, and different levels of Wnt signalling have variable and sometimes opposing effects on cell physiology [4,5]. Thus, while moderate levels of constitutive Wnt activity in CRC cells support cell proliferation, the hyperactivation of Wnt activity by butyrate, a fermentation product of dietary fibre, promotes apoptosis and represses CRC cell growth [4,5]. This effect of butyrate may explain the protective role of fibre against CRC [4,5]. According to these findings, the hyperactivation of Wnt signalling in butyrate-exposed neoplastic colonic cells is required for the protective role of fibre against CRC, and any factor that suppresses this hyperactivation counteracts this preventive activity [2,4,5].

Wnt activity is modified by other signalling pathways; for example, Notch signalling, involved in colonic tumourigenesis, interacts with Wnt activity at several levels [6,7]. In intestinal neoplasms, Notch activity represses Wnt signalling, resulting in down-regulation of Wnt signalling-targeted genes [6]. Conversely, in intestinal precursors, Notch signalling markedly up-regulates proliferation, and this effect requires Wnt activity; further, the presence of both Wnt and Notch signalling synergistically promotes the formation of intestinal tumours in mouse models of CRC [7]. These findings suggest that Wnt and Notch activity cooperate at the initiation phase of colonic tumourigenesis (*e.g*., adenoma formation), but that the two signalling pathways are antagonistic during neoplastic progression. One possible explanation for the differential effects of Notch activity is that by partially inhibiting deregulated Wnt activity, Notch signalling prevents the hyperactivation of Wnt signalling and subsequent apoptosis in colonic neoplastic cells exposed to butyrate. In the absence of Wnt hyperactivation, moderate levels of Wnt signalling support cell proliferation during neoplastic initiation.

## Progerin

Pre-lamin A normally undergoes cleavage by the enzyme Zmpste24, resulting in removal of a farnesylated residue and production of the mature lamin A protein. Hutchinson–Gilford progeria syndrome (HGPS) is a premature ageing disorder caused by a single nucleotide substitution in the lamin A gene, resulting in aberrant *lamin A* pre-mRNA splicing. This aberrant RNA processing results in a mutant form of lamin A, called progerin, which lacks 50 amino acids, including the Zmpste24 cleavage site [8]. Progerin therefore retains the farnesylated residue that is normally removed from pre-lamin A, and this change contributes to aberrant nuclear architecture and other cellular phenotypic changes associated with progeria [8]. Importantly, progerin also occurs at low levels in healthy individuals of all ages, because of infrequent use of a cryptic splice site, and the levels of progerin increase with age [9].

Progerin expression up-regulates downstream targets of Notch signalling, and the defects in cell differentiation because of progerin expression are similar to those observed in cells with constitutively active Notch signalling [10]. Among the Notch signalling components up-regulated by progerin are HES1 and TLE1, known repressors of Wnt signalling [11]. Levels of progerin are highly elevated in a number of cancer cell lines, and ectopic expression of progerin enhances prostate tumourigenicity in a nude mouse model [12]. Additional findings support a link between increased progerin expression in normal (*i.e*., non-progeria) cells and carcinogenesis. For example, farnesyltransferase inhibitors, which block the farnesylation of progerin and have been used in clinical trials for HGPS, have anti-cancer activity [13]. In addition, in renal cell carcinomas, inactivation of the von Hippel-Lindau gene promotes progerin expression, leading to suppressed p53 activity and enhanced cancer cell survival [14]. Progerin is also expressed in human leukaemia cells, further suggesting that progerin expression is linked to age-related carcinogenesis [14].

It has been firmly established that Notch–Wnt crosstalk, which takes place in normal colonic cells, is deregulated in CRC [6,7]. This crosstalk typically involves downstream events in these signalling pathways, such as the repression of Wnt transcriptional activation by the Notch targets HES1 and TLE1 [11]. Thus, colon-specific Notch–Wnt crosstalk is expected to be omnipresent in CRC and independent of upstream regulatory events (*e.g*., Wnt ligand expression and secretion). The role of progerin in promoting deregulated Wnt–Notch crosstalk in CRC has not yet been established. However, progerin expression is known to increase with age [9], and has been identified in prostate cancer cells [12]. Furthermore, expression of lamin A is down-regulated in CRC [15], and enhanced progerin expression can be associated with down-regulation of lamin A (because of alternative RNA splicing). Thus, we posit that with increased age, there is enhanced progerin expression in colonic cells, and these higher progerin levels contribute to the Notch–Wnt signalling crosstalk associated with CRC. There are several mouse models for progeria, including mice deficient in Zmpste24. Treatment of Zmpste24-deficient mice with butyrate improved ageing-related phenotypes and increased lifespan [16]. This finding suggests that butyrate, which augments Wnt activity and likely mediates the protective role of dietary fibre [4,5], can also influence the pathological cell physiology caused by progerin. Thus, butyrate both induces CRC cell apoptosis and has beneficial effects on pro-senescent progeria phenotypes. Whether these effects of butyrate are in part mediated by Wnt–Notch signalling interactions requires future investigation.

**Fig. 1:**
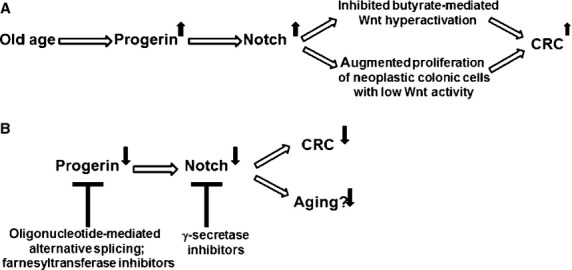
(**A**) Summary of proposed mechanism. Levels of progerin increase with normal ageing, and can enhance Notch signalling, inducing expression of the Wnt inhibitors HES1 and TLE1. This may interfere with the hyperactivation of Wnt signalling by butyrate derived from dietary fibre. At the same time, the enhanced Notch activity promoted by progerin augments proliferation of neoplastic colonic cells with low levels of Wnt signalling. Thus, we hypothesize that progerin, associated with cancer promotion in several tissues, has a net pro-tumourigenic effect on CRC, at least in part through the suppression of Wnt hyperactivation. This suppression would increase as progerin levels rise with age, strengthening the link between ageing and CRC. (**B**) Possible therapeutic approaches against progerin activity may influence age-related CRC risk. Up and down solid arrows represent up-regulation and down-regulation, respectively.

Thus, in summary, we hypothesize that the higher levels of progerin that are associated with age influence Wnt–Notch signalling crosstalk and increase CRC risk. We further posit that increased progerin expression affects the ability of butyrate to repress colonic carcinogenesis, blocking the preventive activity of fibre against CRC. Therefore, a possible mechanism by which, progerin promotes intestinal neoplasia is through preventing the hyperactivation of Wnt signalling by fibre-derived butyrate (Fig. 1A). This inhibition of Wnt hyperactivation could occur through enhanced expression of the Notch targets HES1 and TLE, which repress Wnt signalling [11]. The net effect of inhibiting Wnt hyperactivation would be the maintenance of low levels of deregulated Wnt activity that are required for adenoma formation. Furthermore, these lower levels of Wnt activity can synergize with progerin-induced Notch signalling to enhance colonic tumourigenesis. This latter scenario is supported by findings that reveal cooperative action between Notch signalling and low levels of constitutive Wnt activity in adenoma formation [7].

Therefore, progerin-mediated Notch activity may promote CRC through two interrelated mechanisms. First, Notch signalling targets (*i.e*., HES1 and TLE1) partially repress Wnt activity and inhibit the up-regulation of Wnt signalling by butyrate, thus down-regulating apoptosis of neoplastic colonic cells. At the same time, the lower level of Wnt activity sustained in the presence of HES1/TLE1 can engage in signalling crosstalk with increased Notch activity [7], supporting the proliferation of neoplastic colonic cells. Thus, the higher levels of progerin in normal ageing cells may maintain Wnt–Notch crosstalk that increases CRC risk *via* enhanced adenoma formation and progression to carcinoma.

According to our hypothesis, the protective role of fibre diminishes with age, as progerin activates Notch signalling and blocks Wnt hyperactivation. If this is the case, then approaches to down-regulate progerin Notch signalling in older individuals may enhance the protective role of fibre against CRC.

## Klotho

The transmembrane form of klotho is a coreceptor for fibroblast growth factor-23, which controls serum levels of phosphate and vitamin D [17]. Secreted klotho, consisting of the protein's extracellular domain, acts as a humoural factor [18] that mediates anti-ageing effects and modulates Wnt signalling [18,19]. Mice homozygous for *Klotho* hypomorphic alleles are a model for human ageing, and they exhibit osteoporosis, atherosclerosis, emphysema and infertility [20]. Conversely, mice overexpressing wild-type klotho exhibit an approximate 20% increase in lifespan [18,19]. These *in vivo* data strongly support a role for klotho in cellular senescence and ageing. In agreement with these findings, newborns and the elderly in three human populations exhibit significant differences in the frequencies of *Klotho* gene variants [21]. Individuals homozygous for the *Klotho* allele variant KL-VS were shown to be underrepresented in the elderly population, suggesting an association between this variant and a shortened human lifespan [21].

Of particular interest for CRC is the relationship between klotho, Wnt signalling and ageing. *Klotho* mutant mice exhibit fewer numbers of Brdu-retaining stem cells and increased senescence-associated endogenous β-galactosidase (SAβ-gal) activity [20]. The increased SAβ-gal activity is observed in intestinal crypts, where Wnt activity is usually at its highest levels, and immunoprecipitation assays detected Wnt3-klotho protein complexes [20]. Increasing amounts of klotho inhibit Wnt activity in a dose-dependent fashion, and this effect is dependent upon the klotho-Wnt ligand association [20]. Since klotho does not influence Wnt activity induced by exogenous expression of intracellular beta-catenin, the effects of klotho on Wnt signalling are upstream of beta-catenin, interfering with Wnt ligand-receptor interactions at the plasma membrane [20].

Studies of klotho, and the role of Wnt ligands in CRC, support a role for a colon-specific regulation of klotho–Wnt crosstalk being important in colonic tumourigenesis. Thus, it has been established that the secretion of Wnt ligands is not only important for maintenance of the stem cell compartment in the normal colon, but is also required to maintain pro-proliferative levels of Wnt activity in CRC cells that already possess Wnt pathway mutations [22]. An important Wnt ligand in the colon, and one overexpressed in CRC, is Wnt3a [22], and klotho can associate with Wnt3 to repress Wnt signalling activity. It is known that klotho expression is down-regulated in CRC [23,24]. These findings strongly support a role for klotho in influencing Wnt activity in the normal colon and in colonic tumourigenesis, *via* interactions with Wnt3 and other Wnt ligands, the expression of which are deregulated in CRC [22].

The role of klotho as an inhibitor of Wnt signalling at the ligand level has implications for colonic neoplasia, particularly for the modulation of Wnt signalling and colonic cell physiology by fibre-derived butyrate. Although most cases of CRC are initiated by Wnt pathway mutations resulting in increased levels of transcriptionally active beta-catenin, butyrate up-regulates Wnt activity and apoptosis in colonic cells in part by stimulating Wnt signalling upstream of beta-catenin, at the ligand-receptor level [5]. Thus, Wnt hyperactivation in butyrate-exposed CRC cells may be counteracted by the association of klotho with Wnt ligands. By inhibiting Wnt activity at the ligand level, klotho would interfere with butyrate-mediated Wnt hyperactivation and suppress the preventive activity of butyrate.

Conversely, because of promoter hypermethylation, *Klotho* expression is frequently downregulated in CRC cells compared to normal tissue [23]. This down-regulation of klotho is observed in ageing rhesus monkeys [25], and a similar change in expression might take place during normal human ageing. The resulting lower levels of klotho may increase basal Wnt activity in normal cells, and promote neoplastic intestinal cell proliferation that is dependent upon moderate levels of Wnt signalling activity.

**Fig. 2:**
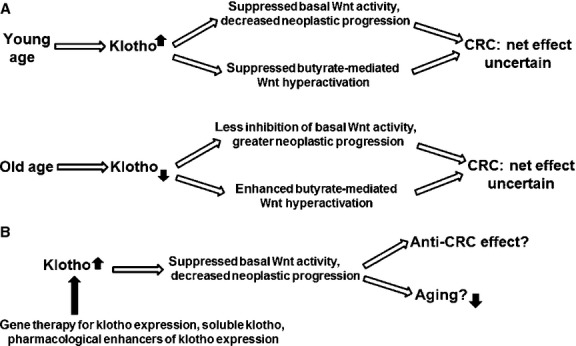
(**A**) Summary of proposed mechanism. The net effects of klotho, typically considered a tumour suppressor, on CRC risk may be complex. Klotho represses basal Wnt activity and thus may lower neoplastic progression. On the other hand, klotho may inhibit butyrate-mediated Wnt hyperactivation, interfering with the chemopreventive action of butyrate. The relative effects of klotho on suppression of basal Wnt activity *versus* suppression of Wnt hyperactivation may depend on which variant of klotho is present. (**B**) Possible therapeutic approaches against klotho activity that can influence age-related CRC risk. Up and down solid arrows represent up-regulation and down-regulation, respectively.

Based upon these reports, we hypothesize that klotho both negatively and positively influences colonic tumourigenesis (Fig. 2A). Klotho may repress colonic tumourigenesis by sequestering Wnt ligands and counteracting basal Wnt activity in colonic neoplasms. Thus, if expression of klotho decreases with age, this may promote Wnt-mediated colonic neoplasia. However, klotho may also suppress the ability of fibre-derived butyrate to hyperactivate Wnt signalling in CRC cells and induce apoptosis. In conclusion, klotho may be anti-tumourigenic based upon its effects on basal Wnt activity, but it may promote CRC through its inhibitory action on butyrate-mediated Wnt hyperactivation and apoptosis.

The latter effect of klotho is plausible since butyrate in part up-regulates Wnt signalling through activity at the ligand level [5], similar to the mechanism by which klotho affects Wnt signalling [20]. Wnt ligand-receptor interactions are important for the maintenance of Wnt signalling in colonic cells, even in CRC cells that have downstream activating mutations in the canonical Wnt pathway [22]. Thus, klotho can repress the ability of fibre/butyrate to hyperactivate Wnt signalling at the plasma membrane level, and this activity of klotho could decrease the preventive activity of fibre against CRC.

What determines whether changes in the expression of klotho have a positive or negative effect on colonic carcinogenesis? It is important to stress that the physiological consequences of Wnt signalling depend upon its levels in colonic cells: whereas moderate Wnt signalling enhances proliferation, hyperinduction of this signalling promotes apoptosis [4,5]. By repressing basal Wnt activity below levels that promote proliferation, klotho may have an anti-CRC effect. However, by inhibiting the pro-apoptotic hyperactivation of Wnt activity in butyrate-exposed CRC cells, klotho may have a pro-tumourigenic effect. Thus, the pro- or anti-tumourigenic effect of klotho in the colon may depend upon several factors, including the levels of butyrate present.

In addition, since *Klotho* gene variants are associated with differential survival in three human populations [21], it is possible that these gene variations exert their effect on lifespan in part by affecting cancer risk. Thus, the underrepresentation of the KL-VS variant in the elderly population might be because of the increased cancer-related mortality of individuals with this variant. We therefore hypothesize that *Klotho* gene variants are under- or over-represented in CRC patients dependent upon how each variant affects basal Wnt signalling and its hyperactivation by fibre-derived butyrate. In this manner, the type of *Klotho* variant present, and its relative expression, can interact with levels of butyrate derived from diet, modifying CRC risk. At the same time, differences in *Klotho* variant expression can influence longevity, resulting in associations between age, cancer risk and lifespan. If levels of klotho decrease with age, the effects on CRC may depend upon which *Klotho* gene variant is present, as well as the specific effects of each variant on basal and butyrate-mediated Wnt activity.

## mTOR and rapamycin

The mammalian target of rapamycin (mTOR), a serine/threonine kinase, is an essential component of the signalling complexes mTORC1 and mTORC2. Both complexes are overexpressed in CRC [26] and play a role in cell growth, proliferation and motility. Activity of mTORC1, but not mTORC2, is inhibited by rapamycin, a natural product derived from the bacterial species *Streptomyces hygroscopicus*, and this inhibition induces cell cycle arrest and apoptosis in cancer cell lines [27]. Importantly, mTOR signalling may also affect human ageing [28]. Since a number of metabolic pathways involved in ageing converge on mTOR signalling, rapamycin and its derivatives have been proposed as an approach to inhibit ageing and prolong lifespan [28]. Thus, mTOR signalling may represent a nodal point linking pro-senescence and pro-tumourigenic cellular pathways; inhibition of mTOR activity may in part prevent cancer associated with age-related deregulation of cell signalling.

The mTORC1 pathway is activated in intestinal polyps of APCΔ716 mice, a murine model of *familial adenomatous polyposis* and sporadic CRC, and this activation is repressed by RAD001, an mTOR inhibitor and rapamycin derivative [27]. RAD001 repression of mTORC1 activity inhibits adenoma formation in APCΔ716 mice, resulting in decreased mortality [27]. Activation of mTORC1 in APCΔ716 mice is supported by Wnt signalling, and, congruently, beta-catenin knockdown in SW480 CRC cells *in vitro* results in reduced mTOR levels and activity [27]. Thus, Wnt signalling up-regulates mTORC1 activity, which in turn enhances the ability of deregulated Wnt activity to promote adenoma formation.

In another model of intestinal cancer, APC(Min/+) mice, rapamycin also inhibits neoplasia and enhances survival [29]. However in control (not treated) APC(Min/+) mice, beta-catenin levels are elevated in adenomas, rapamycin treatment reduces these levels, suggesting that mTORC1 activity can feedback to control levels of Wnt signalling [29]. Finally, in another mouse model of sporadic CRC, rapamycin causes tumour regression in the presence of *Apc* mutation alone, but not in mice with dual *Apc* and *Kras* mutations [30]. These findings indicate a role for the mTORC1 pathway in Wnt signalling-positive CRC and suggest that mTORC1 inhibitors may be effective for CRC treatment [31]. Furthermore, since *Kras* mutation usually occurs later in the colonic neoplastic process, the finding that rapamycin is effective in *Apc*, but not *Apc/Kras* mutant mice [30], suggests that mTORC1 activity is most important at the earlier stages of colonic neoplastic development. Since butyrate is also most effective against early stage neoplasms [4,5], the crosstalk between mTOR activity and butyrate-mediated Wnt signalling might be important in early colonic tumourigenesis (*i.e*., adenoma formation, the risk for which typically increases with age).

mTORC2 inhibition may also be an effective strategy against CRC. Thus, CRC cell lines can be classified as rapamycin sensitive (exhibiting repressed growth when exposed to the agent), and rapamycin resistant [26]. Knockdown of the mTORC2 protein Rictor decreases proliferation of both rapamycin sensitive and resistant CRC cells [26]. Inhibition of mTORC1 by rapamycin induces cell survival AKT signalling and, therefore, results in resistance to therapeutic approaches based upon mTORC1 repression [26]. mTORC2, however, is not affected by rapamycin, and the inhibition of mTORC2 may bypass AKT-mediated rapamycin resistance. Therefore, both mTORC1 and mTORC2 represent potential targets of therapeutic approaches for both ageing and CRC.

**Fig. 3:**
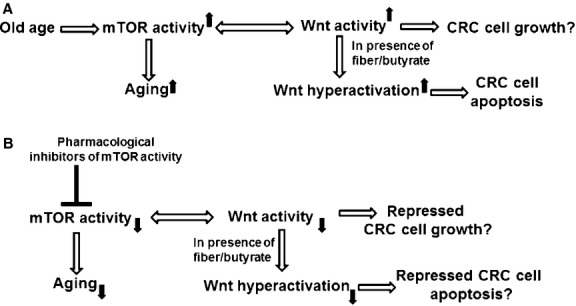
(**A**) Summary of proposed mechanism. Wnt signalling can up-regulate mTOR activity, which likely enhances Wnt activity-mediated tumourigenesis; mTOR activity may also promote ageing and senescence. (**B**) Possible therapeutic approaches against mTOR activity that can influence age-related CRC risk. Up and down solid arrows represent up-regulation and down-regulation, respectively.

We posit that aberrant/deregulated mTOR signalling occurs at greater frequency with increased age, and that this enhanced mTOR activity promotes both cellular senescence [26] and initiation/progression of colonic neoplasia [27,29–31], thus linking ageing with increased CRC risk (Fig. 3A). The importance of colon-specific crosstalk between mTOR and Wnt signalling is underscored by the finding that a wide variety of key components of mTOR signalling are overexpressed in CRC, and differ by cancer stage [32]. Bi-directional crosstalk between mTOR and Wnt signalling in the intestinal tract has also clearly been demonstrated in mouse models of CRC [27–30]. Animal studies suggest that Kras signalling may interfere with mTOR–Wnt crosstalk [30], which may not occur outside the GI tract. The specific mechanisms underlying tissue-specific regulation of mTOR signalling crosstalk remain to be determined.

The relationship between butyrate-induced Wnt hyperactivation, mTOR signalling, and CRC is uncertain. Increased Wnt signalling by butyrate may enhance mTORC1 activity, which is pro-tumourigenic; however, at the same time, Wnt hyperactivation promotes CRC cell apoptosis. Both mTOR activity and butyrate presumably exert their greatest influence at early stage colonic neoplastic development [4,5,30]; therefore, a possible preventive approach against CRC would be to combine Wnt hyperactivation by butyrate with pharmacological suppression of mTOR signalling.

## Chemoprevention, age, and CRC

Some of the age-related signalling pathways discussed here are predicted to decrease the protective role of fibre/butyrate against CRC. Interestingly, this protective effect might be most powerful against right-sided colonic neoplasms, since the highest levels of fibre-derived butyrate have been detected in the right colon [33–35]. Therefore, interactions between age-associated signalling pathways and effects of butyrate on Wnt signalling may predominantly take place in the right colon, and may explain the reported increase in the ratio of right/left colon carcinomas with age [36,37]. The proximal shift of colonic neoplasms with ageing is unlikely to be related to the relative decrease in left neoplasms because of screening by sigmoidoscopy, since the observations of the trend were made prior to the increase in sigmoidoscopy screening rates. Furthermore, the ratio of right/left colon adenomas (the precursors of colonic neoplasms) also increases with age: the ratio is 0.59 for 20–49 years old individuals and 0.81 for 50–89 years old individuals [38].

Colorectal cancer is considered age-related cancer, and therefore, the US Preventive Services Task Force recommends screening for CRC beginning at age of 50. However, a recent study reported a significant increase in CRC incidence among 20- to 34-year olds; according to this report, if the current trend continues, by 2030 we will see up to 124% increase in CRC incidence in this age group [39]. This shift in age of diagnosis is most likely also associated with the lower protective effect of butyrate against CRC; however, in young CRC patients that loss of protection could be explained with the consumption of a diet low in fibre; whereas, in older patients, the loss of protection by butyrate could be because of increase in age-associated signalling as discussed here. In the very young CRC patients, the putative powerful effect of a highly aberrant diet may enhance CRC risk independent of the influence of age-related signalling (*e.g*., progerin, klotho, mTOR).

In addition to fibre, the intake of non-steroidal anti-inflammatory drugs, calcium and vitamin D supplements is associated with reduced incidence of CRC [40,41]; how these chemopreventive agents interact with age-related signalling pathways is unknown. Further exploration of these interactions can result in age-customized CRC preventive strategies.

## Age, CRC and potential therapeutics

Based on the hypotheses outlined here, a number of possible therapeutic approaches can be proposed. With respect to progerin (Fig. 1B), it has been shown that an oligonucleotide-based approach can modify *lamin A* pre-mRNA splicing and thus down-regulate expression of progerin, restoring a more normal phenotype and gene expression pattern to HGPS cells [42]. A similar gene therapy approach may be beneficial against cancers affected by age-related progerin expression; in addition, this approach would have the benefit of enhancing lamin A expression, which is down-regulated in CRC [15]. According to our hypothesis, down-regulation of progerin would release the inhibition of butyrate-mediated Wnt hyperactivation and apoptosis; thus enhancing the preventive efficacy of dietary fibre (and the therapeutic efficacy of histone deacetylase inhibitors) against CRC. In addition, farnesyltransferase inhibitors, which block the farnesylation of progerin, have anti-cancer activity [13], and may also down-regulate the downstream effects of progerin on Notch signalling. Inhibition of Notch signalling by γ-secretase inhibitors [43] is another therapeutic option that would interfere with Wnt-Notch crosstalk, and block the downstream effects of progerin expression.

Klotho expression may have either positive or negative effects on colonic tumourigenesis (Fig. 2A); however, since klotho deficiency is linked to enhanced ageing [17,18,20,19,21] and to chronic kidney disease (CKD) [44], and since klotho expression is down-regulated in CRC [23,24], therapeutic approaches to up-regulate klotho expression are more likely to be adopted. In animal models of CKD, virus-mediated gene delivery has successfully enhanced klotho expression, and direct administration of soluble klotho protein in animal models has been shown to repress Wnt signalling and enhance longevity [[44] and references therein]. In addition, *in vitro* studies have identified a number of pharmacological agents capable of up-regulating klotho expression [45], which can be further tested for *in vivo* efficacy. Ultimately, these approaches should be evaluated in animal models of CRC to determine whether (*i*) enhanced klotho expression suppresses Wnt activity in intestinal neoplasia and thus reduces tumour burden, and (*ii*) the reduced Wnt activity resulting from klotho expression interferes with the chemopreventive action of butyrate.

Given the importance of mTOR signalling in cancer (Fig. 3), a number of inhibitors of this pathway are under study [26–32,46]. Thus, pharmacological inhibition of mTOR signalling, utilizing agents already in clinical use, clinical trial or animal experiments, is the most likely approach for interfering with the putative role of mTOR signalling in age-related CRC risk. As with progerin and klotho, close attention must be paid to how repression of mTOR activity affects both basal Wnt activity, as well as butyrate-mediated Wnt hyperactivation. Furthermore, similar to progerin and klotho, approaches that target mTOR signalling may have beneficial effects on the human lifespan independent of potential effects on tumour formation.

## Future directions

*In vitro* CRC cell culture experiments can evaluate the validity of our hypotheses by ascertaining the effects of progerin and klotho variant expression, as well as altered mTOR signalling, on butyrate-induced Wnt hyperactivation and apoptosis. Analyses of CRC patient samples can determine whether increased expression of progerin, and changes in the expression of klotho and its variants, are associated with increased risk of CRC. It would be of considerable interest to determine whether progerin levels differ between normal *versus* neoplastic human colonic tissues, and whether different *Klotho* gene variants are associated with altered CRC risk and/or with the age at which cancer is diagnosed. Microarray analyses can identify genes and signalling pathways responsible for these effects. Data generated by these analyses will identify targets that modulate both ageing and CRC.

*In vitro* studies and patient sample analyses should be followed up by *in vivo* experiments. If, for example, a *Klotho* variant is found to modulate Wnt activity *in vitro*, and this variant is differentially expressed in human normal and neoplastic tissues, the role of the variant can be addressed with transgenic mouse models. In addition, both *Klotho* mice homozygous for hypomorphic alleles of *Klotho* [20], as well as *Klotho*-overexpressing mice [18,19], can be crossed to *Apc* mutant mice to obtain *Klotho* repression or overexpression in the *Apc* mutant background. Response to butyrate with respect to tumour burden and lifespan will then be evaluated. Similar experiments can be performed with mice overexpressing a relevant variant form of the *Klotho* gene, to ascertain how different forms of klotho that are found in humans influences intestinal neoplasia. *Apc* mutant mice can also be crossed with progeria model mice [47,48] to determine effects of progerin on tumour burden, and on the modulation of tumour burden by butyrate. Similarly, the effect of rapamycin on the modulation of tumour burden by butyrate can be evaluated in *Apc* mutant mice. If progerin-Apc or klotho-Apc hybrid mice exhibit altered tumour burden compared to *Apc* mutant mice, one can ask whether treatment of the hybrid mice with rapamycin influences the effects of progerin and klotho on intestinal tumourigenesis. The various approaches discussed above, in our section on potential therapeutics, can be tested in animal models of CRC and their efficacy evaluated, particularly in conjunction with fibre/butyrate.

## Conclusion

The incidence of CRC and most other cancers increases with age. While this is in part because of accumulation of mutations with time, mechanisms directly linking processes of cellular senescence/human ageing with initiation of intestinal neoplasia may also play a role in increased risk of CRC with age. The progerin, klotho and mTOR pathways are linked to senescence/ageing, and influence signalling pathways pivotal to the development of CRC. Determination of the role of klotho, progerin and mTOR in colonic carcinogenesis will be a fundamental advance in our understanding of cancer as part of the human ageing process. Novel therapeutic strategies focused on klotho, progerin and mTOR signalling have the potential to both enhance longevity and repress cancer associated with old age.
